# Comparison of the Macro Chain Transfer Agent and the Macro Azo Initiator Based on the Poly(3-hydroxy Butyrate) in the Polymerization Kinetics of Methyl Methacrylate

**DOI:** 10.1021/acsomega.4c08996

**Published:** 2025-02-06

**Authors:** Baki Hazer, Özgür Keleş

**Affiliations:** † Department of Aircraft Airframe Engine Maintenance, Kapadokya University, Ürgüp, Nevşehir 50420, Turkey; ‡ Department of Chemistry, Bülent Ecevit University, 67100 Zonguldak, Turkey; § Department of Mechanical Engineering and Engineering Science, University of North Carolina at Charlotte, Charlotte, North Carolina 28223, United States

## Abstract

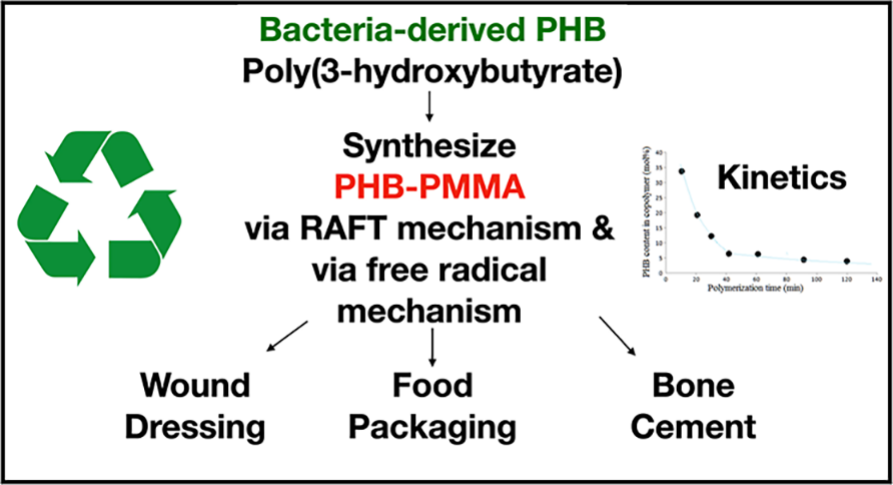

Poly(3-hydroxybutyrate) (PHB) derivatives are attractive for sustainable polymer production, yet their role in controlling radical polymerization kinetics remains underexplored. In this study, we compare the polymerization kinetics of methyl methacrylate (MMA) using two PHB-based macroinitiators: a macro chain transfer agent (PHB-macro reversible addition-fragmentation chain transfer (RAFT)) and a macroazo initiator (PHBai). RAFT polymerizations (PHB-R-PMMA) were conducted at 70 °C with PHB-macro RAFT in the presence of 2,2′-azobis(isobutyronitrile), while conventional free radical polymerizations (PHBaiPMMA) were carried out using PHBai under identical conditions. The RAFT system exhibited a slightly lower overall rate constant (*k* = 1.11 × 10^–4^ L/mol·s) compared to the azo-initiated system (*k* = 1.28 × 10^–4^ L/mol·s). Both systems showed a gradual decrease in the PHB content over time, indicating effective copolymer formation with increasing MMA incorporation. Activation energies for PHB-macro RAFT and PHBai were calculated as 0.88 and 1.05 kJ/mol, respectively, demonstrating RAFT’s superior control over molecular architecture. The resulting PHB-PMMA block copolymers offer promising applications in orthopedic surgery (e.g., bone cements), packaging, medical implants, drug delivery, and dental materials. This study provides the first direct comparison of PHB-based macro RAFT and azo systems for MMA polymerization, highlighting RAFT’s advantage in achieving controlled polymer architectures and expanding biomedical and industrial utility.

## 1 Introduction

Nonbiodegradable plastics have been a major source of environmental pollution. The growing concerns about environmental degradation and the finite nature of fossil fuel resources have heightened interest in biodegradable polymers. Among these, poly(3-hydroxyalkanoates) (PHAs) stand out due to their natural production and accumulation in certain bacteria under limited growth conditions. Poly(3-hydroxybutyrate) (PHB), a biodegradable and hydrophobic member of the PHA family, offers a sustainable alternative to traditional, nondegradable plastics. PHB presents promising applications in various polymer industries (e.g., orthopedic surgery as bone cements, packaging materials, wound dressings, and medical implants such as surgical sutures and dental implants).^1−8^

Poly(3-hydroxybutyrate) (PHB) was first discovered in 1926 by Maurice Lemoigne as an intracellular polymer synthesized by bacteria such as *Ralstonia eutropha* and *Pseudomonas putida* under nutrient-limited conditions with excess carbon sources. Its unique combination of thermoplastic properties, biocompatibility, and biodegradability makes it an attractive material for numerous applications including biomedical devices, food packaging, and agricultural films. However, large-scale commercialization was initially constrained by high production costs and process inefficiencies. Advances in genetic engineering and fermentation technologies, particularly involving *Cupriavidus necator* and engineered strains of *Escherichia coli*, have significantly improved production yields and reduced costs, making PHB a viable alternative to petroleum-derived plastics.^2,5,9−11^ Moreover, recent advances in genome editing technologies, including CRISPR-Cas,^9^ have further enhanced the biosynthetic capabilities of these bacteria, offering new possibilities for large-scale PHB production with tailored properties.^12^

**Figure 1:**
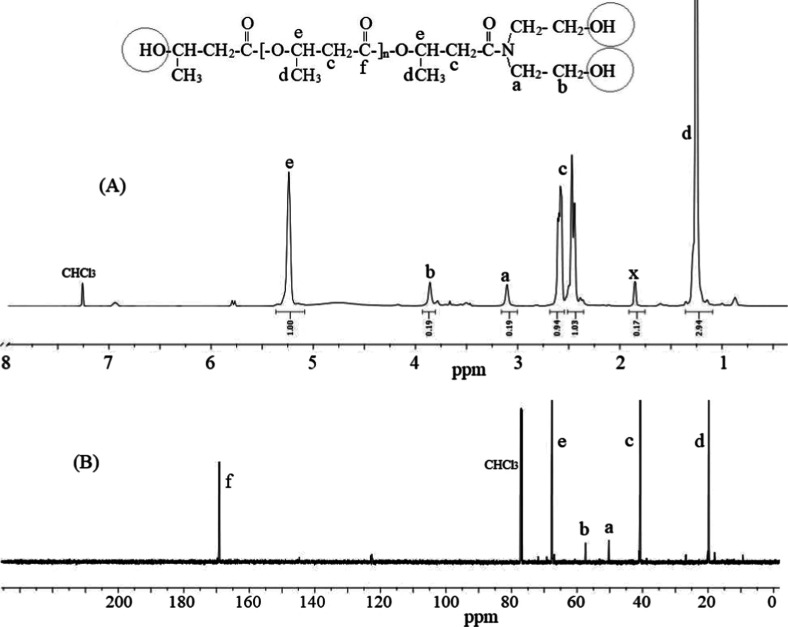
^1^H NMR (A) and ^13^C NMR (B) spectra of the obtained PHB-DEA.

**Figure 2:**
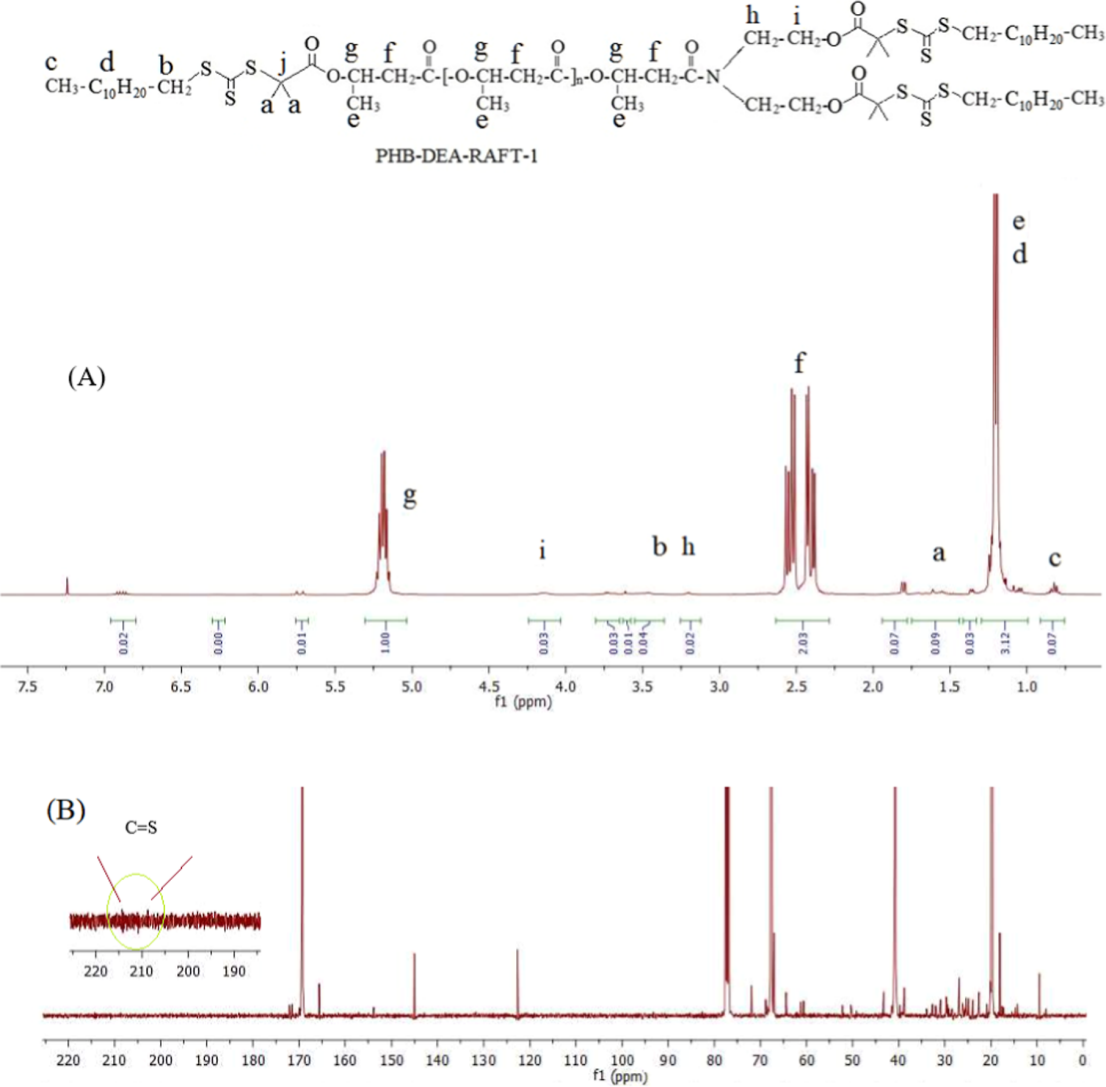
^1^H NMR (A) and ^13^C NMR (B) spectra of the obtained PHB-R.

**Figure 3:**
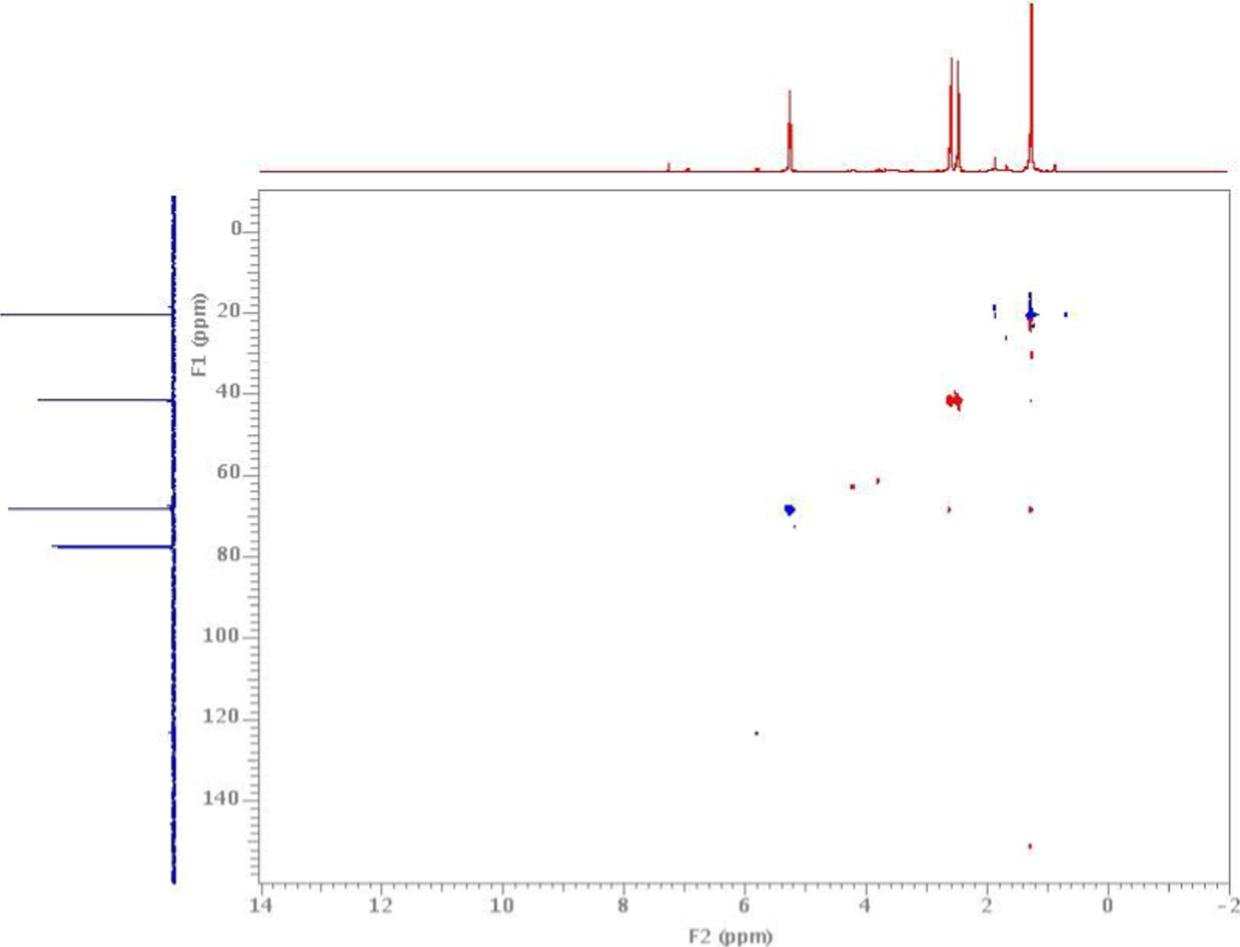
HSQC NMR spectrum of PHB-R.

**Table 1:** RAFT Polymerization of MMA by PHB-R_2_ (MMA: 1.00 g, AIBN: 3 mg, DMF: 1.0 g, 80 °C). Rh (Q): Hydrodynamic Ratio

cop analysis											
code	PHB-R_2_ (g)	time (min)	yield (g)	Ln(Mo/M)	PHB (%)^a^	MMA (g)	[M] (mol/L)	*M*_n_ (Da)	*M*_w_ (kDa)	PDI	Rh (Q) (nm)
PHB-MMA-11	0.10	10	0.116	0.08	33.7	0.077	922	93	124	1.32	1.6
PHB-MMA-12	0.10	20	0.172	0.15	19.6	0.138	862	72	104	1.49	1.4
PHB-MMA-13	0.10	30	0.250	0.25	12.0	0.220	780	84	120	1.43	1.5
PHB-MMA-14	0.10	42	0.351	0.40	6.0	0.330	670	92	125	1.36	1.6
PHB-MMA-15	0.10	60	0.450	0.63	6.4	0.421	530	88	126	1.45	1.7
PHB-MMA-16	0.10	91	0.595	0.83	5.0	0.565	435	80	107	1.33	1.7
PHB-MMA-17	0.10	120	0.715	1.15	4.5	0.683	317	84	111	1.33	1.7

a PHB content was calculated from the ^1^H NMR spectrum.

**Figure 4:**
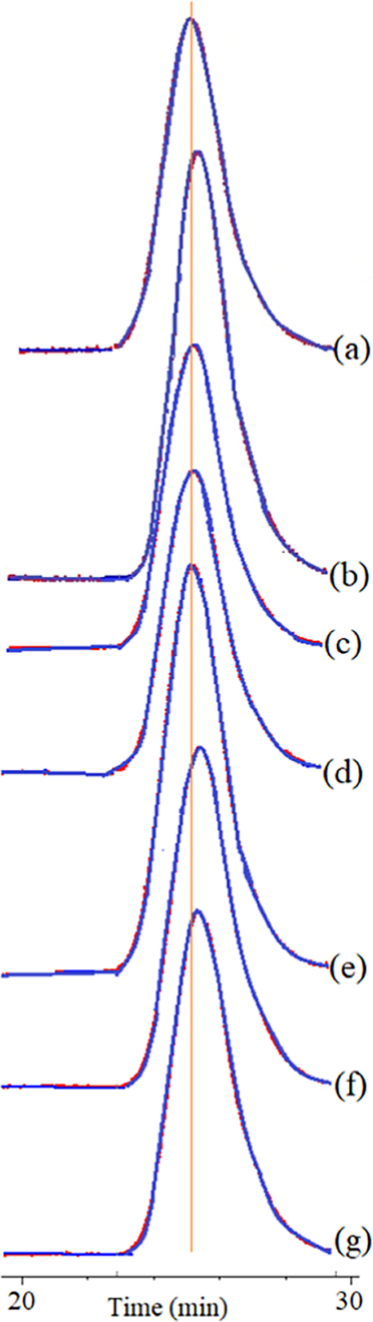
SEC chromatograms of PHB-PMMA block copolymer series: (a) PHB-PMMA-11, (b) PHB-PMMA-12, (c) PHB-PMMA-13, (d) PHB-PMMA-14, (e) PHB-PMMA-15, (f) PHB-PMMA-16, and (g) PHB-PMMA-17.

**Figure 5:**
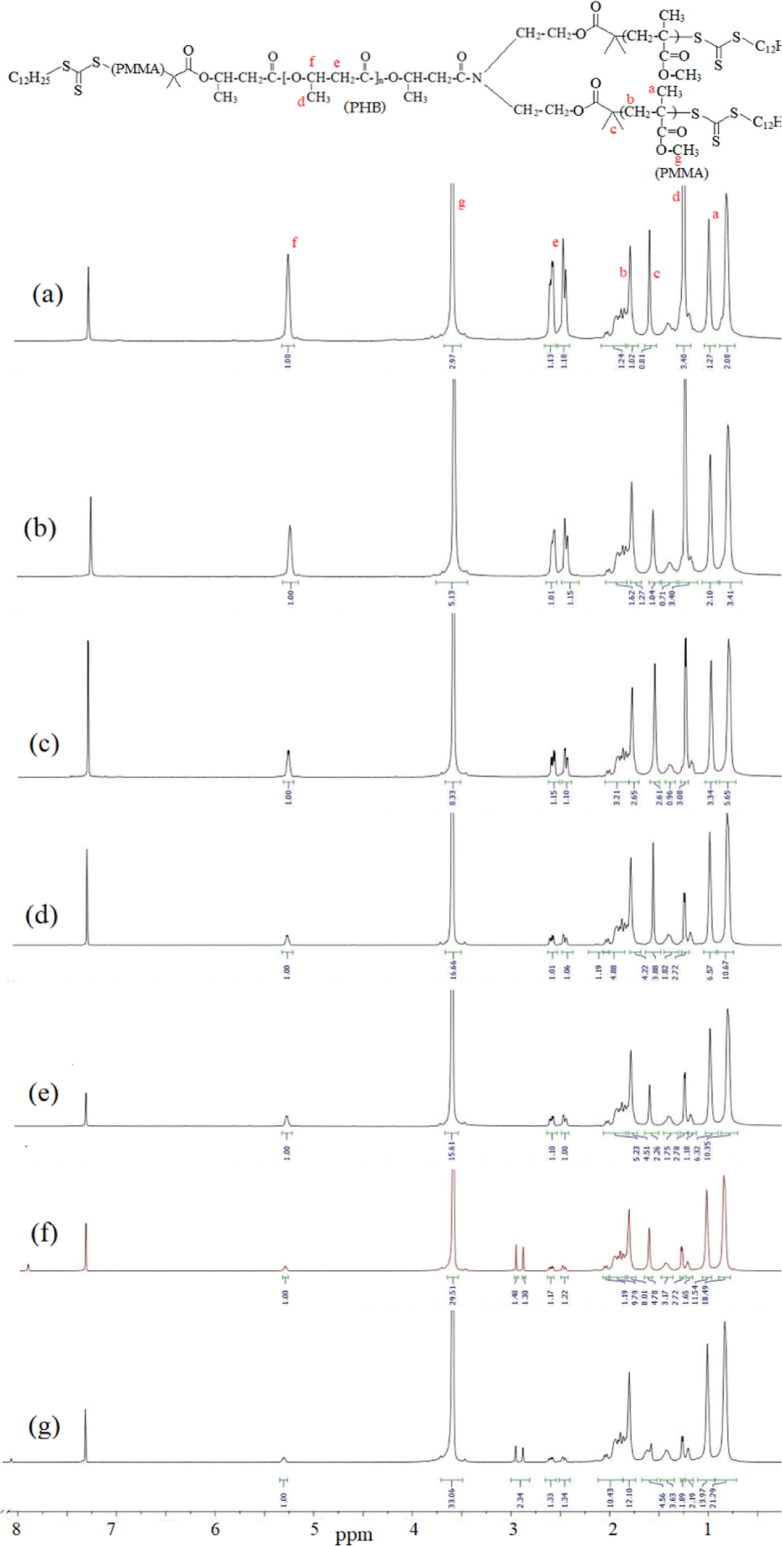
^1^H NMR spectra of the obtained PHB-PMMA block copolymers: (a) PHB-PMMA-11, (a) PHB-PMMA-11, (b) PHB-PMMA-12, (c) PHB-PMMA-13, (d) PHB-PMMA-14, (e) PHB-PMMA-15, (f) PHB-PMMA-16, and (g) PHB-PMMA-17. f Is at 5.2 ppm (−O–CH– in PHB); g is at 3.5 ppm (CH3–O–C– in PMMA).

**Figure 6:**
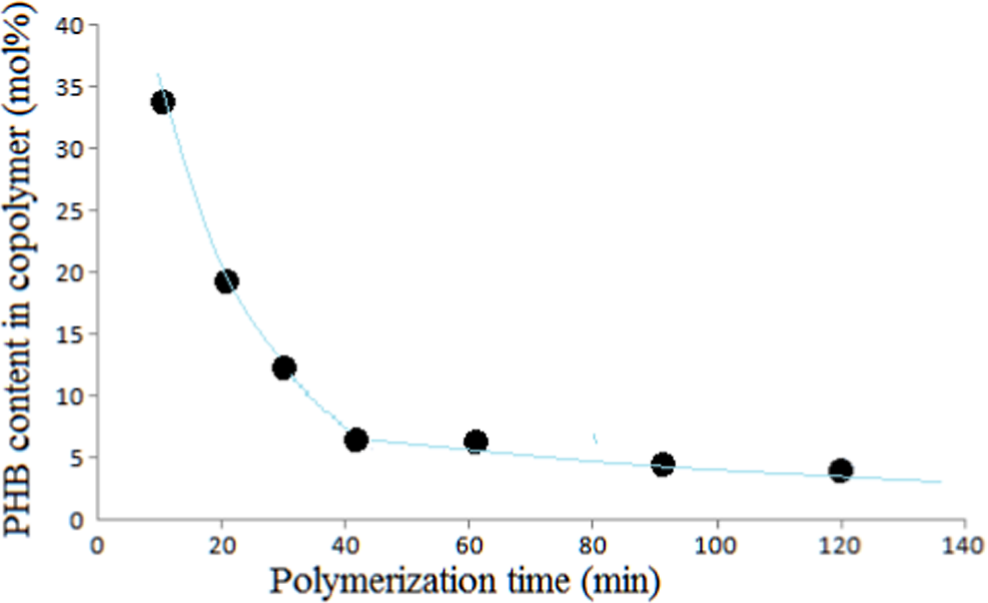
Plot for PHB content in copolymer against polymerization time.

**Figure 7:**
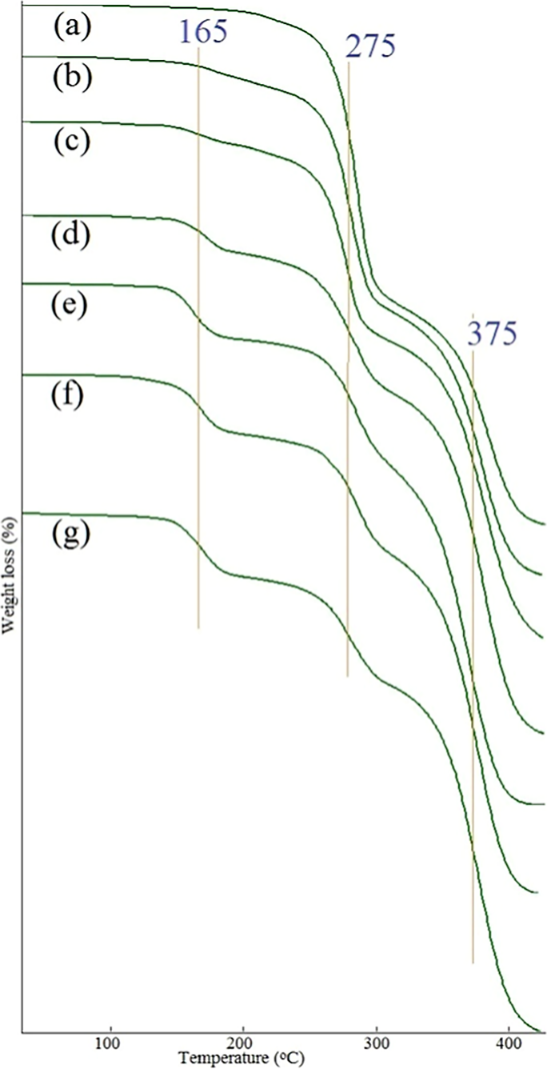
TGA curves of PHB-PMMA block copolymer series: (a) PHB-PMMA-11, (b) PHB-PMMA-12, (c) PHB-PMMA-13, (d) PHB-PMMA-14, (e) PHB-PMMA-15, (f) PHB-PMMA-16, and (g) PHB-PMMA-17. Decomposition at 165 °C is likely attributed to solvent residues or impurities.

**Figure 8:**
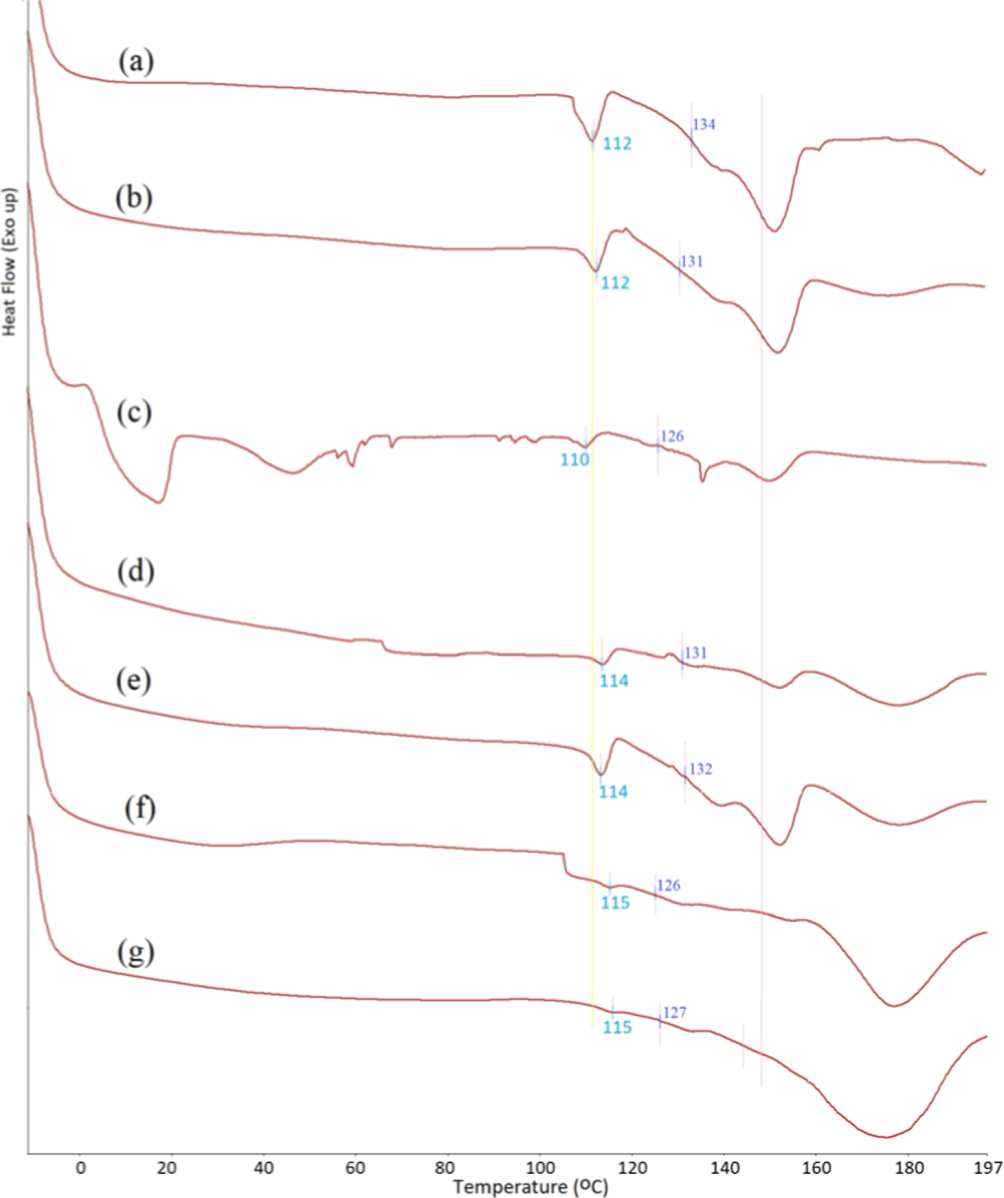
First heating cycles of DSC curves of PHB-PMMA block copolymer series: (a) PHB-PMMA-11, (b) PHB-PMMA-12, (c) PHB-PMMA-13, (d) PHB-PMMA-14, (e) PHB-PMMA-15, (f) PHB-PMMA-16, and (g) PHB-PMMA-17.

**Figure 9:**
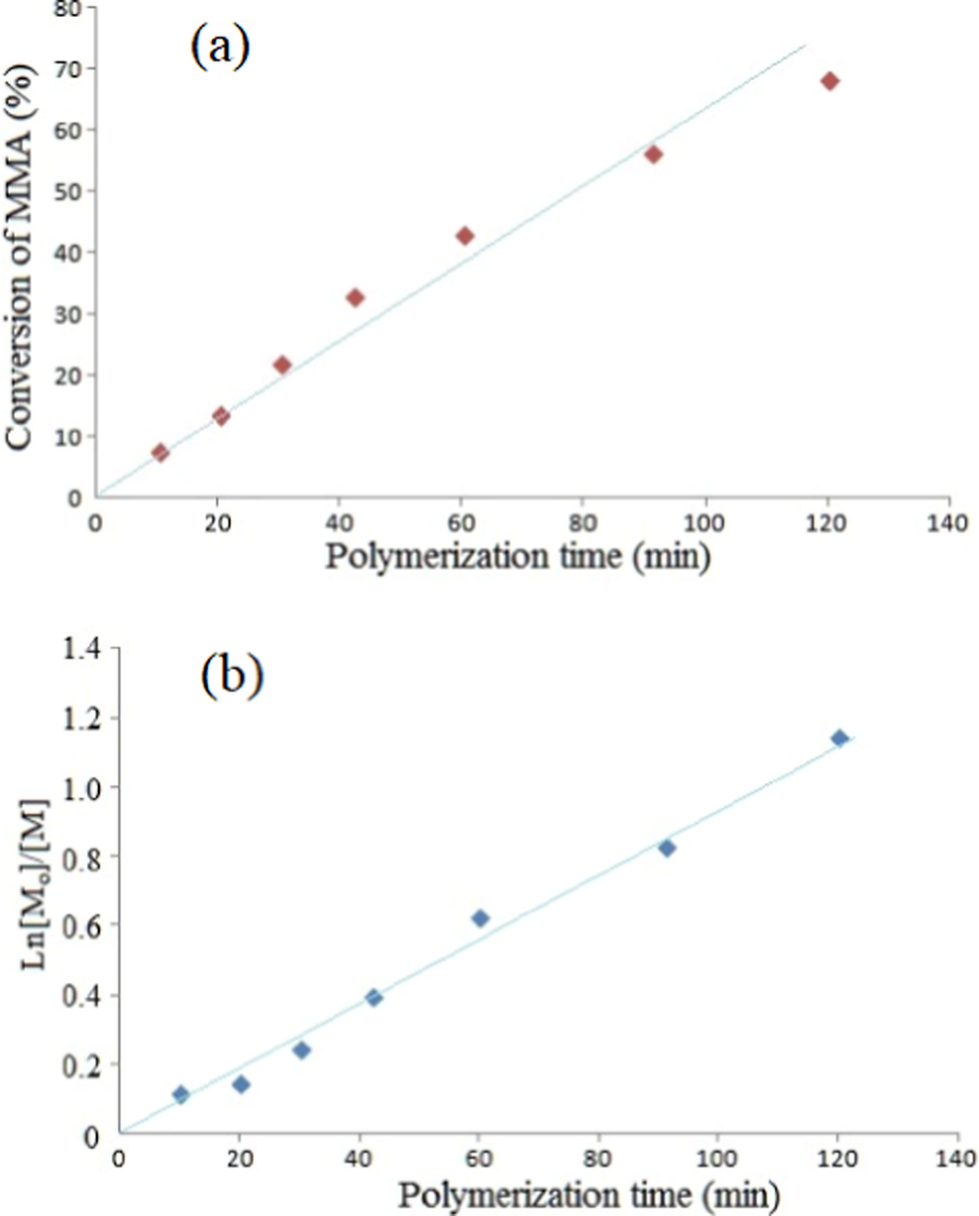
Kinetic plots for the RAFT polymerization of MMA using a PHB-macro RAFT agent at 80 °C: (a) Conversion vs polymerization time (linear fit *R*^2^ = 0.95), (b) Ln [*M*_o_]/[*M*] vs polymerization time (linear fit *R*^2^ = 0.98).

**Figure 10:**
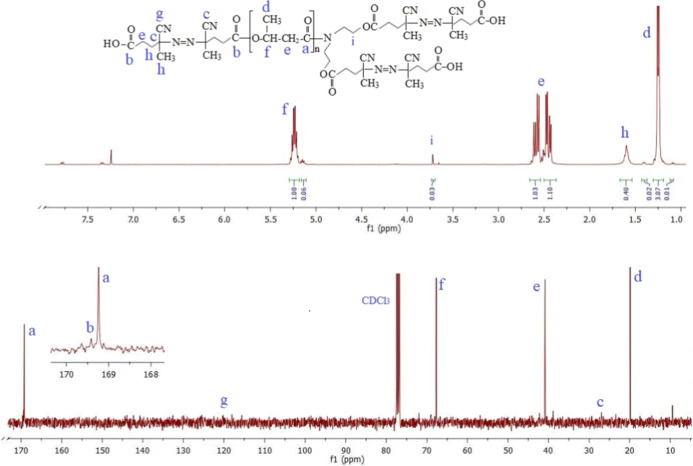
^13^C NMR spectrum of PHBai.

**Figure 11:**
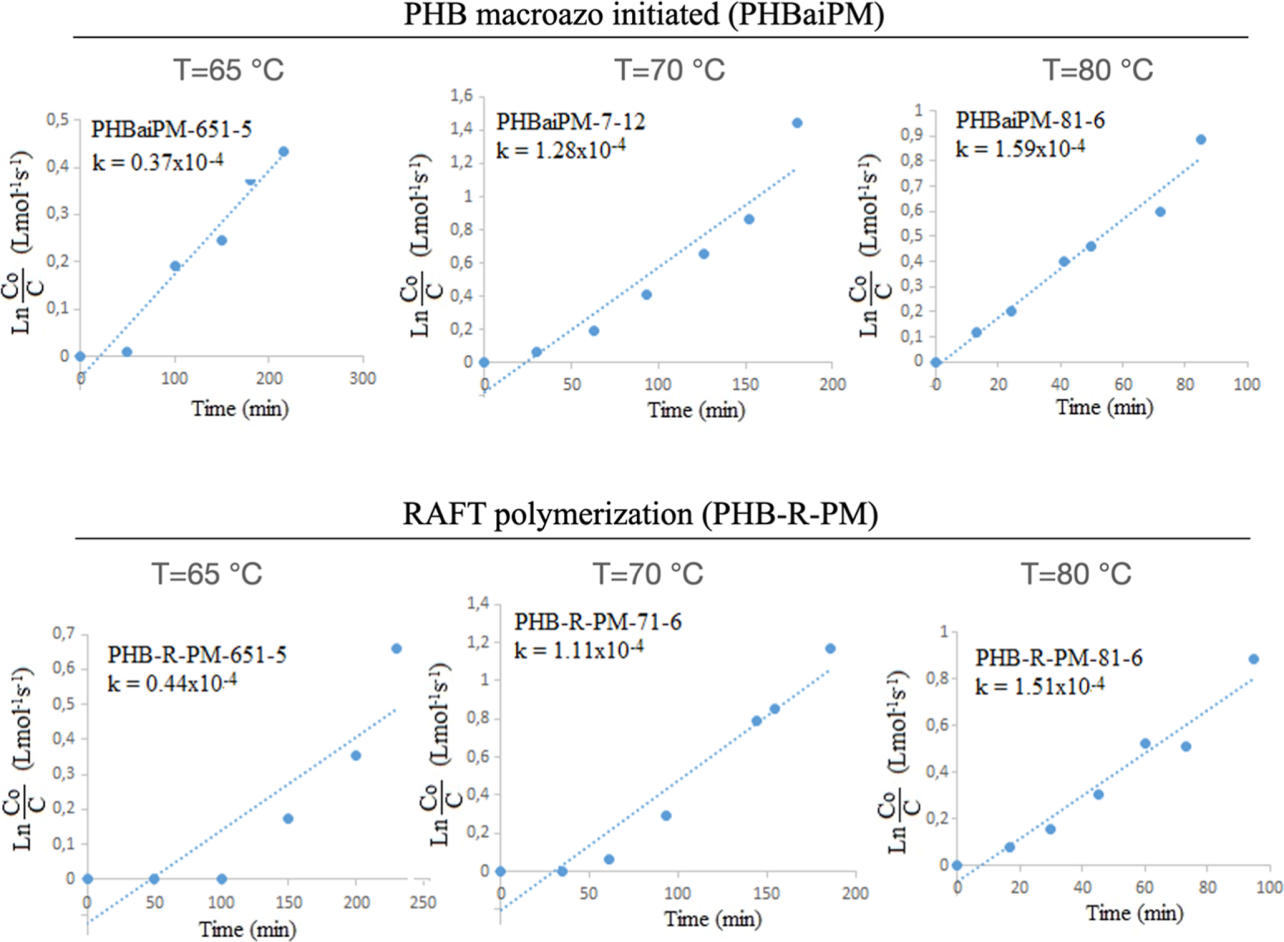
Plots of Ln(*C*_o_/*C*) versus polymerization time for the calculation of the overall rate constant (k) in MMA polymerization. The linear fit *R*^2^ values are: (a) 0.95, (b) 0.96, (c) 0.99, (d) 0.93, (e) 0.94, and (f) 0.99.

**Figure 12:**
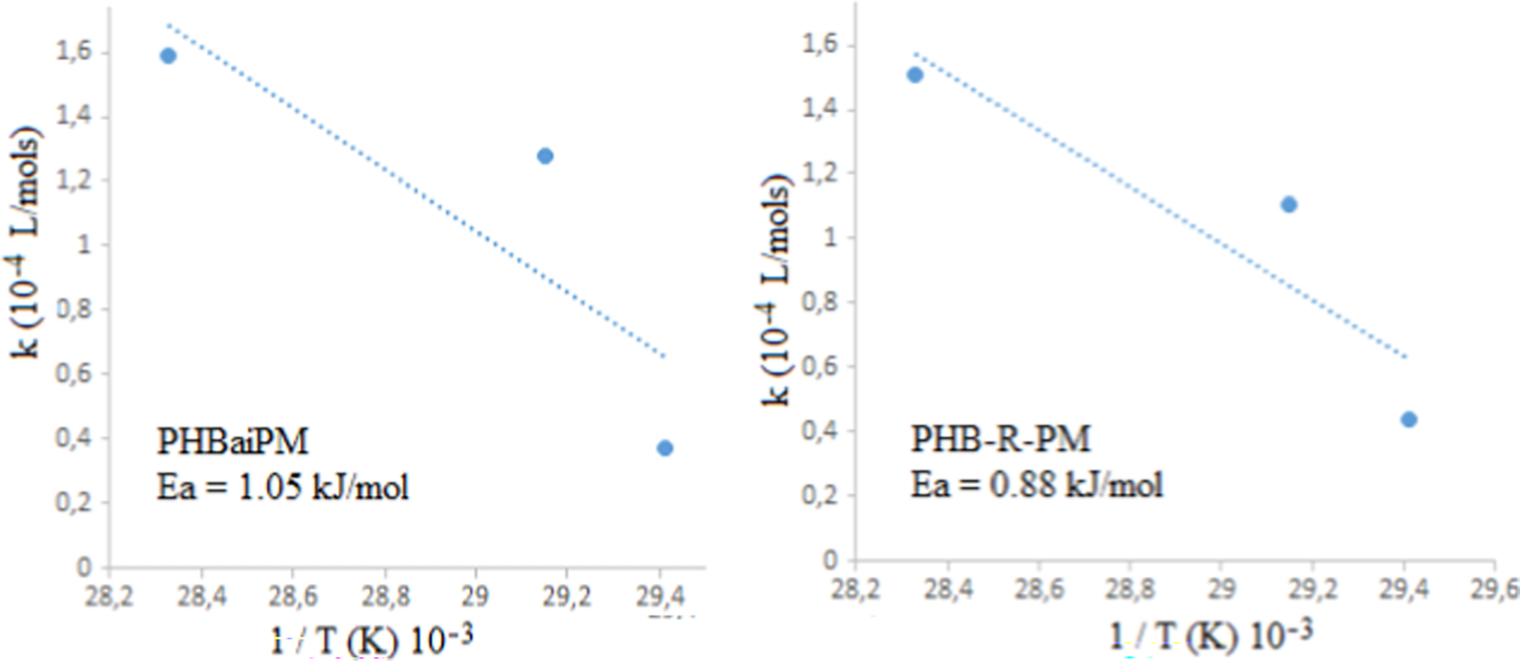
Activation energies of the MMA polymerization with macro intermediates from the Arrhenius equation plotted drawn *k* values against reverse of polymerization temperature. Linear fit *R*^2^ values are (a) 0.71 and (b) 0.82.

The global market for PHB has experienced substantial growth in recent years due to the increasing emphasis on sustainable materials and regulatory initiatives promoting biodegradable plastics. Packaging applications, which capitalize on PHB’s strong hydrolysis resistance and excellent barrier properties, dominate the market. Additional applications include wound dressings and surgical implants, leveraging PHB’s biocompatibility and biodegradability.^5,8,13^ Also, PHB has shown utility in creating nanocomposites for drug delivery and tissue engineering, further extending its biomedical applications.^27^ Recent studies predict the PHB market to grow at a compound annual growth rate exceeding 15% from 2024 to 2030. With its renewable origins and expanding applications, PHB continues to play a crucial role in the development of eco-friendly materials for diverse industries.^6,9,14,15^ Furthermore, PHB has demonstrated promising environmental benefits in biodegradability studies conducted in marine and soil environments, positioning it as a key material for addressing global plastic pollution.^16^

The development of PHB-PMMA block copolymers through reversible addition-fragmentation chain transfer (RAFT) polymerization presents substantial potential across various industrial and biomedical sectors. One prominent application is in orthopedic surgery, where these materials can be used as bone cements, leveraging the biocompatibility of PHB and the mechanical strength of PMMA.^17^ Additionally, these copolymers are promising for packaging materials due to their strong hydrolysis resistance and low gas permeability.^18^ In the biomedical field, PHB-PMMA copolymers can be developed into wound dressings that enhance healing by promoting cell migration and reducing inflammation.^19^ Furthermore, these materials are suitable for use in medical implants, such as surgical sutures and dental implants, due to their biocompatibility and biodegradability.^10,20^ Additionally, PHB-PMMA copolymers can be used in drug delivery systems, benefiting from their ability to form stable nanoparticles^21^ and in the fabrication of dental materials like dentures and fillings, taking advantage of PMMA’s transparency and PHB’s biodegradability.^22^ Overall, the versatile applications of PHB-PMMA block copolymers underscore their significant impact, making them a valuable addition to polymer science and technology.^23^

For these and other industrial applications, PHB needs modification reactions to increase the mechanical, thermal, hydrophilic, and film properties. In this context, here, we reported unique chlorinated PHBs that enable further functionalization.^24−26^ The carboxylic acid end of the PHB was turned into hydroxyl ends reacting with diethylene glycol in the presence of dibutyltin dilaurate, and dihydroxylated PHB (PHB-diol) was obtained.^28,29^ Langlois et al. grafted PHB copolymers with hydroxyethyl methacrylate in the presence of benzoyl peroxide.^30^ Moreover, Loh and co-workers synthesized the PHB-macro initiator by the reaction between PHB-diol and bromoisobutyryl bromide to use atom transfer radical polymerization of *N*-isopropylacrylamide^31^ and *N*,*N*-dimethyl amino ethyl methacrylate.^32^ We recently reported the synthesis of a hydroxylated PHB with the reaction of PHB and diethanolamine. The iodinated radiopaque PHB derivatives were obtained by the reaction between the hydroxyl terminals of the hydroxylated PHB with the iodo benzoic acid moieties.^33^

Methyl methacrylate (MMA) is a biobased monomer, and radical polymerization of acrylates is one of the most widely used processes for the commercial production of high molar mass polymers.^34,35^ Vinyl polymers such as poly(MMA) and PMMA are not biodegradable. It would be very desirable to combine the biodegradability of the biodegradable polyesters and the excellent application properties of polyolefins such as packaging materials, strong hydrolysis resistance, low dielectric constant, transparency, and gas permeability. Living free radical polymerization, such as atom transfer radical polymerization,^36^ nitroxide-mediated polymerization,^37^ or RAFT polymerization,^38−42^ is widely applied for the synthesis of well-defined polymers from a large number of vinylic monomers, including acrylates, acrylamides, methacrylamides, methacrylates, or vinyl esters. Among these polymerization techniques, the robust and versatile nature of RAFT has allowed it to become one of the most useful tools in modern polymer synthesis.^43,44^

Many efforts have been made toward the copolymerization of lactones and vinyl- or vinylidene monomers. Nguyen and Marchessault thermally degraded the natural high molecular weight of PHB to oligomers and esterified it with hydroxyethyl methacrylate to prepare PHB-macromonomer. Atom transfer radical polymerization of MMA with PHB macromonomer with a bromide ATRP initiator led to PHB-PMMA graft copolymer.^45^ A new PHB-macro RAFT agent was used in the controlled living free radical polymerization of *N*-isopropylacrylamide.^20^ Macro azo initiators lead to block/graft copolymers in the free radical polymerization of the vinyl monomers.^46,47^ Macro azo initiators can be synthesized by the reaction of 4,4′-azo bis cyanopentanoic acid with a hydroxyl functionalized polymer (c.a. polyethylene glycol).^48^

In addition to highlighting the broad applications of PHB-PMMA materials, this work specifically examines the polymerization kinetics of macro-RAFT and macro-azo systems. RAFT polymerization is renowned for its “living” characteristics, minimizing termination events and enabling precise control over molecular weight and dispersity. In contrast, azo-initiated free radical polymerizations typically follow conventional kinetics, often resulting in broader molecular weight distribution.^38−40,42,46−48^ Understanding these mechanistic differences is essential for fine-tuning the properties of the resulting copolymers, such as thermal stability, chain architecture, and degradability—attributes critical for biomedical applications where consistent molecular weight and biocompatibility are imperative.

In this work, we report the RAFT polymerization kinetics of MMA initiated by the PHB-macro RAFT agent. PHB-macro RAFT agent was prepared by the modified procedure reported by the published articles. First PHB was refluxed with diethanolamine to obtain three hydroxyl-functionalized PHB. Then the hydroxyl ends were turned to trithio carbonate leading to PHB-macro RAFT agent. MMA was polymerized with the PHB-macro RAFT agent to obtain the PHB-PMMA block copolymer. The combination of PHB with PMMA could have potential applications as a material for orthopedic surgery (c.a., bone cements). Because of the biodegradable PHB block and biobased PMMA, more potential applications would be good to include. Here, we report the RAFT polymerization kinetics of MMA at 80 °C in a dimethylformamide solution. Structural and thermal characterizations of PHB-PMMA were evaluated.

## 2 Experimental Section

### 2.1 Materials

Poly(3-hydroxy butyrate) (PHB), a microbial polyester (*M*_n_ 187,000 g/mol, *M*_w_/*M*_n_ 2.5, Biomer Inc.), was supplied by BIOMER (Germany). Dimethylformamide (DMF), *N*,*N*′-dicyclohexylcarbodiimid (DCC), dimethyl amino pyridine (DMAP), 4,4′-azo bis cyanopentanoic acid, stannous 2-ethyl hexanoate (Sn-oct_2_), diethanol amine (DEA), 2-(dodecylthiocarbonothioylthio)-2-methylpropionic acid (DDMAT is the R_2_), and the other chemicals were purchased from Sigma-Aldrich and used without further purification. MMA and methacrylated polyethylene glycol (*M*_n_ 500 g/mol) were purchased from Sigma-Aldrich, and the inhibitor was removed by passing through basic aluminum oxide before use.

### 2.2 Synthesis of Trihydroxylated PHB (PHB-dea)

For the modified synthesis,^49^ a mixture of 60.3 g of vacuum-dried PHB, 82 g of DEA, and 1.7 g of Sn-oct was refluxed in 180 mL of CHCl_3_ for 2 h. The solvent was distilled under atmospheric conditions (not in the rotary evaporator) up to one-half of the solution. Then the mixture was leached with excess methanol and filtered. The crude product was dried under vacuum at 40 °C. For further purification, the obtained polymer was dissolved in 100 mL of CHCl_3_ and filtered into the excess methanol (300 mL) with stirring in order to precipitate PHB-OH. The pure PHB-DEA was dried under vacuum at 40 °C for 24 h. Yield was 36 g. Characteristic FTIR signals: 1567 cm^–1^ amide carbonyl; 3301 cm^–1^ primary hydroxyl groups of DEA; 1721 cm^–1^ belongs to ester carbonyl of PHB. The characteristic chemical shifts of the PHB-dea sample in ^1^H NMR spectrum were observed at 1.3 ppm for −CH_3_, 2.4–2.6 ppm for −CH_2_–COO–, 3.0 ppm for −N–CH_2_–, 3.5–3.8 ppm for CH_2_–OH, 4.1 ppm for −CH–OH and 5.1–5.3 ppm for −CH–O–.

### 2.3 Synthesis of PHB Macro RAFT Agent (PHB-R)

The PHB macro-RAFT agent (PHB-R) was obtained by the reaction between PHB-DEA and R_2_. For the modified synthesis,^50^ PHB-DEA (4.84 g) was dissolved in CHCl_3_ (30 mL). DCC (2.31 g), R_2_ (0.28 g), and DMAP (0.60 g) were added into this solution continuously stirring at 40 °C for 24 h. The precipitated side product, dicyclohexyl urea, was removed via filtration. The solution was poured into 100 mL of MeOH to precipitate the PHB-macro RAFT agent. For further purification, the crude product was dissolved in CHCl_3_ and reprecipitated from methanol. Yield was 3.82 g. The white solid product was dried under a vacuum at 40 °C for 24 h. The GPC result (in DMF) was *M*_n_ 3800 Da, PDI 1.225. Water uptake was 6% for PHB film and 62% for PHB-OH-27 coarse powder.

### 2.4 Synthesis of PHB Macro Azo Initiator (PHBai)

The PHBai macroazo initiator was synthesized according to ref (50) The reaction was carried out between PHBdea (9.23 g) and 2,2′-azobis cyanopentanoic acid (0.86 g) in the presence of DCC (0.69 g) and DMAP (0.16 g) in CH_2_Cl_2_ (28 mL) and continuously stirred at room temperature for 48 h. The precipitated side product was removed via filtering. The solvent was evaporated up to one-fifth. The excess methanol (ca. 150 mL) was filtered into the concentrated solution to precipitate the macroazo initiator (PHBai), which was dried under vacuum at room temperature, giving a yield of 6.8 g.

### 2.5 RAFT Polymerization of MMA Initiated by PHB-R_2_

RAFT polymerization of MMA was carried out at 80 °C in DMF. For example, the mixture of 0.507 g of PHB-R_2_, 19 mg of AIBN, and 1.22 g of MMA was dissolved in 5 mL of DMF in a glass bottle. Previous studies have shown that PHB readily dissolves in DMF, which has a boiling point of 153 °C, well above 80 °C. Argon was introduced into the solution for 1 min. The RAFT polymerization of MMA using the PHB-macro RAFT initiator was carried out under argon at 80 °C for a given time. Then, the crude polymer solution diluted with 5 mL of CHCl_3_ was precipitated in 200 mL of methanol. The obtained block copolymer samples were dried overnight under a vacuum at 40 °C for 24 h.

### 2.6 Characterization

#### 2.6.1 ^1^H NMR

^1^H and ^13^C NMR spectra of the obtained products in CDCl_3_ solutions were recorded at 25 °C with an Agilent NMR 600 MHz NMR (Agilent, Santa Clara, CA, USA) spectrometer. The RAFT agent (*R*^2^) was purified and sent to the NMR analysis. The NMR spectra of the purified PHB-R_2_ are included in the current study.

#### 2.6.2 Size Exclusion Chromatography Analysis

Molecular weights were determined by size exclusion chromatography (SEC) using a Viscotek GPCmax Auto sampler system, consisting of a pump, three ViscoGEL GPC columns (G2000H HR, G3000H HR and G4000H HR), and a Viscotek differential refractive index detector. The flow rate of the DMF mobile phase was 1.0 mL/min at 30 °C. A calibration curve was generated with five polystyrene (PS) standards of molecular weight 2960, 8450, 50,400, 200,000, and 696,500 Da with low polydispersity. The individual polymer sample solutions containing 0.05 g of THF in 10 mL of THF were filtered and injected automatically into the instrument. Data were analyzed using Viscotek Omni SEC Omni 01 software. The GPC results showed that the extracted PHB had a molecular weight of 187 000 Da (PDI 2.5), while this value dropped to 4700 Da (PDI 1.5) for PHB-DEA after transamidation reaction.

#### 2.6.3 Thermal Analysis

Thermal analysis of the obtained polymers was carried out under nitrogen using TA Q2000 differential scanning calorimetry (DSC) and Q600 Simultaneous DSC—thermogravimetric Analysis (TGA) (SDT) series thermal analysis systems. DSC measures temperatures and heat flows associated with thermal transitions in the polymer samples obtained. The dried sample was heated from −60 to 190 °C under a nitrogen atmosphere at a rate of 10 °C/min. The mass loss of the samples was determined by TGA under a nitrogen atmosphere using a Setaram Labsys Evo 1150 apparatus by heating from 30 to 600 °C at 10 °C min^–1^.

## 3 Results and Discussion

Natural PHB was refluxed with DEA in chloroform and distilled at 95 °C. After the hydroxylation reaction, the obtained product was dissolved in chloroform, filtered from the insoluble part, and precipitated from excess amount of methanol. The insoluble fraction was at around at 30 wt %, which is in good agreement with the synthesis of telechelic diols.^28,51^ During this work, PHB-DEA and PHB-R_2_ macroinitiators were obtained at least 6 times with the same characteristic signals in NMR spectra. The characteristic signals of PHB and DEA parts in PHB-DEA were observed in ^1^H and ^13^C NMR spectra. Figure 1 shows the ^1^H NMR (A) and ^13^C NMR (B) spectra of the obtained PHB-DEA. As the molar mass decreases, the characteristic NMR signals of DEA (a and b) come out taller.

The PHB-R_2_ macro RAFT agent was synthesized by esterifying hydroxyl-terminated PHB-DEA with R_2_ catalyzed by DCC and DMAP. The crude product was purified by filtering off byproducts and precipitating the polymer in methanol. The ^1^H NMR spectrum (Figure 2A) shows characteristic resonances for PHB at ∼1.25 ppm (methyl protons, −CH_3_), ∼2.5 ppm (methylene protons, −CH_2_–C(O)−), and ∼5.25 ppm (methine proton, −CH–O−). Peaks attributed to the RAFT end-group functionality appear at ∼3.1 ppm (−N–CH_2_–O−) and ∼3.9 ppm (−O–CH_2_–O−), consistent with the trithiocarbonate group. Additional signals from the dodecyl chain of R_2_ are observed at ∼3.25 ppm (−CH_2_–S−), ∼1.70 ppm (−C(CH_3_)_2_−), ∼1.25 ppm (−(CH_2_)_10_−), and ∼0.85 ppm (terminal −CH_3_). The chloroform peak is at ∼7.26 ppm.

Minor or unassigned signals near 5.8 ppm (^1^H, Figures 1A and 2B) and 126 and 146 ppm (^13^C, Figures 1B and 2B) likely originate from crotonate-type byproducts, commonly observed in PHB due to partial degradation. Oligomerization of PHB during the reaction may lead to some side reaction as crotonates formation with double bonds according to refs (8 and 52). Weak peaks around 1.6–1.8 ppm are attributed to overlapping methylene protons, trace impurities, or residual solvents. These data confirm the successful incorporation of the RAFT moiety, validating PHB-R_2_ as a macro RAFT agent suitable for controlled radical polymerization.

In the ^13^C NMR spectrum of PHB-R_2_ (Figure 2B), the PHB backbone exhibits characteristic resonances at ∼20 ppm (methyl carbons, −CH_3_), ∼40 ppm (methylene carbons, −CH_2_–C(O)−), ∼67 ppm (methine carbons, −CH–O−), and ∼169 ppm (ester carbonyl carbons, −C=O), consistent with typical poly(3-hydroxybutyrate) assignments. Signals at ∼52 ppm (−N–CH_2_–O−) and ∼58 ppm (−O–CH_2_–O−) correspond to the diethanolamine-derived linkages retained from the PHB-DEA intermediate. The successful incorporation of the trithiocarbonate (RAFT) functionality is confirmed by a resonance at ∼185 ppm (thiocarbonyl carbon, −C=S) from DDMAT (R_2_). Additionally, 78 ppm is attributed to the CDCl_3_ solvent. Together, these resonances validate the integrity of the PHB segments and the successful functionalization with the RAFT moiety, confirming the synthesis of the PHB-R_2_ macro RAFT agent.

^1^H–^13^C Heteronuclear Single Quantum Coherence Spectroscopy (HSQC) shows which hydrogens are directly attached to which carbon atoms. Furthermore, HSQC NMR provides additional insight into the spectroscopic correlations. All of the C/H correlations for PHB-R_2_ are established using both 1H and 13C NMR spectra by means of HSQC NMR technique (Figure 3). In this manner, C/H correlations of PHB-R_2_ (F1_ppm_) at C 67.2 and 5.25; at C 40.2 and 2.5; at C 58.0 and 3.75; at C 60.2 and 4.22; at C 26.1 and 1.62; and at C 20.2 and 1.80/1.27/0.85 were all observed.

### 3.1 Synthesis of PHB-PMMA Block Copolymers

RAFT copolymerization of MMA was performed using a PHB-R macro RAFT agent at 80 °C in DMF solution. The polymerization conditions and results are given in Table 1. A series of PHB-PMMA block copolymers were obtained in different polymerization times while keeping the amounts of macro RAFT agent (R_2_), monomer (MMA), initiator (AIBN), and the solvent (DMF) constant. A smooth increase in polymer yield was observed as polymerization time increased. SEC was used to determine molar masses of the PHB-PMMA block copolymer series. The whole SEC chromatograms were unimodal, as shown in Figure 4. Slight changes in molar masses were observed by the time. The molar masses of the obtained block copolymers were ranged from 93 to 83.7 kDa. RAFT polymerization leads to a polymer with a polydispersity of 1.1 at low conversions. However, polydispersity of the obtained PHB-PMMA block copolymers was between from 1.32 to 1.49. Some limitations of RAFT polymerization have also been reported by published articles.^53−56^

The hydrodynamic ratio of the polymer samples was also determined by the SEC instrument at around 1.4 to 1.7. The structural characterization of the PHB-PMMA block copolymers was done using their ^1^H NMR spectra containing characteristic signals of PHB and PMMA blocks. Figure 5 shows the ^1^H NMR spectra of the obtained PHB-PMMA block copolymers, comparatively. The integral ratio of the signal at 5.2 ppm (−O–CH– in PHB) to the signal at 3.6 ppm (−COOCH_3_, PMMA) in the NMR spectrum rendered the PHB content (%) in the PHB-PMMA block copolymer. Table 1 contains the calculated PHB contents from the ^1^H NMR spectra of the block copolymer series. PHB content of the copolymer was proportional to the monomer MMA concentration in feeding. Decrease in PHB content as the polymerization time increases can be seen in Figure 6.

### 3.2 Thermal Properties

TGA and DSC were used to characterize the thermal properties of the PHB–PMMA block copolymers. TGA resulted in decomposition temperatures for each segment in the obtained block copolymers. Figure 7 shows the TGA curves of the PHB-PMMA block copolymer series. The DTG plot is the derivative of the TGA plot. So, TGA and DTG show the same decomposition temperatures. A series of the block copolymers decomposed in three steps. The most volatile parts decomposed first at 165 °C. This probably belongs to the methyl ester side component of the PMMA segment. Typical decomposition of the PHB blocks was observed at the middle temperature at 275 °C. Finally, PMMA blocks decomposed at 375 °C.

Figure 8 shows the DSC curves of the PHB-PMMA block copolymer series, in which we see the glass transition temperatures (*T*_g_) and melting transition temperatures (*T*_m_) of the polymers. *T*_ms_ of the PHB blocks were seen at temperatures (112–115 °C) lower than that of the natural PHB (170 °C). The low molar mass of the PHB segments in the obtained block copolymers can cause this lower temperature. PMMA segments show the typical *T*_g_ at around 130 °C same as that of the PMMA homopolymer. After glass transition of PMMA, the polymer begins to soften and then melts.

### 3.3 Polymerization Kinetics

PHB-PMMA block copolymer series were obtained starting the polymerization time from 10 to 120 min while keeping the concentrations of RAFT agent, AIBN, and MMA constant at 80 °C. Conversion of the monomer was the linearly proportional to the polymerization time, but for the longer durations, it tends to deviate from the linearity (Figure 9 a). It is well-known that the molar masses of the obtained polymer increase by the time in RAFT polymerization.^57^ Interestingly, PHB-PMMA-12, −13, and −14 obey this rule, which means that molar masses gradually increase from 72 to 92 kDa. Then, the molar masses of the following polymers stand in 80–87 kDa, which may be described as the increase in molar mass ending after a while shortly. Plot of Ln [*M*_o_]/[*M*] vs polymerization time was linear and the calculated rate constant was 1.61 × 10^–4^ s^–1^. The rate constant for RAFT polymerization of NIPAM using the PHB-macro RAFT agent at 70 °C (*k* = 6.86 × 10^–4^ s^–1^) is higher than that for MMA, indicating that MMA has lower monomer reactivity compared to NIPAM.^58^

Perrier and Takolpuckdee reviewed the RAFT polymerization kinetics of some acrylates using the molecular chain transfer agents and xanthates at 60 °C with the overall rate constant 10^–3^ L/mols without solvent.^55^ Using the macro PHB-RAFT agent, polymerization overall rate constants were found to be lower than these values, even at a polymerization temperature of 80 °C. The use of solvent and macroRAFT agent could cause a slower polymerization rate.

### 3.4 Comparison of the Activation Energies of the PHB Macro RAFT Agent and PHBai in the MMA Polymerization

Synthesis of PHBai: Hydroxylated PHB was reacted with 4,4′-azo bis cyano pentanoic acid in the presence of DCC and DMAP at room temperature to obtain PHB macroazo initiator (PHBai). The ^1^H NMR spectrum of PHBai and the ^13^C NMR spectrum of PHBai were approved for structural confirmation. ^1^H NMR, f1_ppm_ 5.2 (−OCH−), 3.7 (−CH_2_–CH_2_–O−), 2.5 (−CH_2_–C(O)−), 1.6 (−CH_2_–C–, CH_3_–C−), 1.2 (CH_3_–CH−). ^13^C NMR, f1_ppm_ 20 (CH_3_–CH−), 27 (−C–CN), 40 (−CH2–C(O)), 67 (−O–CH−), 120 (−CN), 169.2 (−C(O) for PHB), and 169.3 (−C(O) for ai) in Figure 10. Existence of the characteristic azo bis cyanopentanoic acid functional groups was observed in the NMR spectra, agreeing with refs (44 and 59).

Copolymer series were prepared at 65, 70, and 80 °C to study the polymerization kinetics by means of the calculation of the activation energies using the reaction rate constants. As shown in Figure 11, all plots drawn in between Ln(*C*_o_/*C*) and polymerization time were straight lines, so the slope gave the *k* value. As expected, as polymerization temperature increases, overall rate constants (*k*) increases for both the azo initiator and the RAFT agent. The polymerization kinetics, as depicted in Figure 11, reveal distinct trends, depending on the temperature. At 65 °C, the rate constant (*k*-value) for RAFT polymerization (PHB-R-PM, 0.44 × 10^–4^ L/mol·s) is higher than that of the azo-initiated system (PHBaiPM, 0.37 × 10^–4^ L/mol·s). At 70 °C, however, the azo-initiated system exhibits a slightly higher rate constant (1.28 × 10^–4^ L/mol·s) compared to RAFT polymerization (1.11 × 10^–4^ L/mol·s). Similarly, at 80 °C, the azo initiator again shows faster kinetics (1.59 × 10^–4^ L/mol·s) relative to RAFT polymerization (1.51 × 10^–4^ L/mol·s). These observations indicate that while azo-initiated systems generally demonstrate higher reactivity at elevated temperatures, RAFT polymerization exhibits comparable performance at lower temperatures (e.g., 65 °C), emphasizing its robustness and suitability for controlled polymerization under milder conditions.

The kinetic evaluation of RAFT polymerization and FRP demonstrates distinct behaviors influenced by polymerization mechanisms. RAFT polymerization consistently produced PHB-PMMA block copolymers with narrower polydispersity indices (PDIs), reflecting their controlled nature. For example, at 80 °C, RAFT polymerization yielded PDIs between 1.32 and 1.49, indicating narrow molecular weight distributions. In contrast, FRP exhibited higher PDIs, indicative of broader molecular weight distributions and less controlled polymerization. These differences arise from the living characteristics of RAFT polymerization, where active chain transfer agents mediate radical activity, minimizing termination events and ensuring uniform polymer growth.

The implications of these kinetic differences are evident in the copolymers’ thermal and structural properties. While the glass transition temperatures (*T*_g_) of the PMMA segments, approximately 130 °C, were consistent across both methods, the RAFT-derived copolymers demonstrated an enhanced thermal stability. For instance, TGA revealed that the decomposition temperatures of PMMA segments were higher in RAFT samples compared with FRP, likely due to fewer defects and more uniform block distributions. These findings highlight RAFT polymerization’s ability to achieve superior control over molecular architecture, translating into improved thermal and physical properties, which are crucial for high-performance applications such as biomedical devices and advanced packaging.

Activation energies of the MMA polymerization with macro intermediates were calculated from the Arrhenius equation plotting drawn *k*-values against the inverse of the polymerization temperature, as shown in Figure 12. The lowest energy barrier is referred to as the activation energy. It can be concluded that RAFT polymerization requires a slightly lower activation energy (0.88 kJ/mol) compared to free radical polymerization (1.05 kJ/mol). This difference highlights RAFT polymerization’s ability to initiate and sustain polymerization more efficiently under milder conditions, which is critical for preserving the integrity of sensitive polymer components such as PHB. Additionally, the lower activation energy facilitates greater control over polymer chain growth, leading to copolymers with narrower molecular weight distributions and enhanced uniformity. These factors contribute to the superior thermal stability and mechanical properties of RAFT-synthesized copolymers, making this method particularly advantageous for high-performance applications in biomedical devices and advanced packaging.

## 4 Conclusions

Poly(3-hydroxybutyrate) (PHB)-based derivatives are increasingly recognized for their potential in sustainable polymer production due to their biodegradability and tunable properties. This study provided a direct comparison of RAFT and free radical polymerization kinetics using PHB-based macroinitiators, highlighting their distinct roles in achieving controlled polymer architectures. The findings underscore the versatility of PHB derivatives in the production of advanced materials for biomedical and industrial applications. The following conclusions can be made based on the experimental data:

1. RAFT polymerization using PHB-macro RAFT agents exhibited near first-order kinetics (*k* ≈ 1.50 × 10^–4^ L/mol·s), producing copolymers with molecular weights of 80–93 kDa and moderate polydispersity indices (PDIs 1.32–1.49).
2. Conventional free radical polymerization with PHB-azo initiators demonstrated slightly higher rate constants at elevated temperatures but resulted in broader molecular weight distributions.
3. TGA revealed a distinct three-step decomposition pattern for PHB-PMMA copolymers, with PHB blocks decomposing around 275 °C and PMMA blocks at 375 °C.
4. DSC indicated melting transitions for PHB segments at 112–115 °C and glass transitions for PMMA segments at ∼130–131 °C.
5. ^1^H and ^13^C NMR spectroscopy confirmed effective copolymer formation, validating the presence of both PHB and PMMA segments.
6. The activation energy for RAFT polymerization (0.88 kJ/mol) was lower than that for azo-initiated polymerization (1.05 kJ/mol), underscoring RAFT’s superior control over molecular architecture under milder conditions.

This comparative study highlights the advantages of RAFT-mediated PHB-PMMA synthesis including enhanced polymerization control and uniform molecular weight distribution. These findings provide valuable insights into designing advanced PHB-based materials with tunable properties, paving the way for future research on scalable RAFT systems with functionalized quantum dots toward unique nanocomposites.^60^ The combination of biodegradable PHB and mechanically robust PMMA holds promise for applications such as orthopedic surgery (e.g., bone cements), medical implants, packaging, and wound dressings, especially in scenarios requiring precise molecular weight control and thermal stability.

## Data Availability

The original contributions presented in the study are included in the article/ Supplementary material; further inquiries can be directed to the corresponding author.
